# Androgen Activity Is Associated With PD-L1 Downregulation in Thyroid Cancer

**DOI:** 10.3389/fcell.2021.663130

**Published:** 2021-08-06

**Authors:** Timmy J. O’Connell, Sina Dadafarin, Melanie Jones, Tomás Rodríguez, Anvita Gupta, Edward Shin, Augustine Moscatello, Codrin Iacob, Humayun Islam, Raj K. Tiwari, Jan Geliebter

**Affiliations:** ^1^Department of Pathology, Microbiology and Immunology, New York Medical College, Valhalla, NY, United States; ^2^United States Military Academy Preparatory School, West Point, NY, United States; ^3^RNA Therapeutics Institute, University of Massachusetts Medical School, Worcester, MA, United States; ^4^Medical Scientist Training Program, University of Massachusetts Medical School, Worcester, MA, United States; ^5^Department of Otolaryngology, New York Eye and Ear Infirmary, New York, NY, United States; ^6^Department of Otolaryngology, New York Medical College, Valhalla, NY, United States; ^7^Department of Pathology, New York Eye and Ear Infirmary, New York, NY, United States

**Keywords:** PD-L1 pathway, androgen receptor, immune surveillance, gender disparity, thyroid cancer

## Abstract

Thyroid cancer is the most prevalent endocrine malignancy in the United States with greater than 53,000 new cases in 2020. There is a significant gender disparity in disease incidence as well, with women developing thyroid cancer three times more often than men; however, the underlying cause of this disparity is poorly understood. Using RNA-sequencing, we profiled the immune landscape of papillary thyroid cancer (PTC) and identified a significant inverse correlation between androgen receptor (AR) levels and the immune checkpoint molecule PD-L1. The expression of PD-L1 was then measured in an androgen responsive-thyroid cancer cell line. Dihydrotestosterone (DHT) treatment resulted in significant reduction in surface PD-L1 expression in a time and dose-dependent manner. To determine if androgen-mediated PD-L1 downregulation was AR-dependent, we treated cells with flutamide, a selective AR antagonist, and prior to DHT treatment to pharmacologically inhibit AR-induced signaling. This resulted in *a* > 90% restoration of cell surface PD-L1 expression, suggesting a potential role for AR activity in PD-L1 regulation. Investigation into the AR binding sites showed AR activation impacts NF-kB signaling by increasing IkBα and by possibly preventing NF-kB translocation into the nucleus, reducing PD-L1 promoter activation. This study provides evidence of sex-hormone mediated regulation of immune checkpoint molecules *in vitro* with potential ramification for immunotherapies.

## Introduction

Thyroid cancer is the most common endocrine cancer and accounts for 3.8% of all cancers (Nguyen et al., 2015). According to the Surveillance, Epidemiology, and End Results Program (SEER), the incidence of thyroid cancer has increased by threefold over the past three decades (Kilfoy et al., 2009) though stabilized during this past decade (Morris et al., 2016). Of the 53,000 new thyroid cancer cases that occurred in 2020, approximately 40,000 occurred in women and 13,000 occurred in men (Siegel et al., 2020). Age-adjusted SEER incidence rates show a three to fourfold increase in incidence of thyroid cancer in women aged 20–49 as compared to men aged 20–49. A study of 566 papillary thyroid cancer (PTC) patients similarly concluded that women have a higher incidence of PTC than males, and also found that men tended to present with larger carcinomas (Yorke et al., 2016). Previously, it was shown that female gender is associated with a high prevalence of RET/PTC1 and RET/PTC3 fusions in PTC (Su et al., 2016). Changes in estrogen receptor (ER) and androgen receptor (AR) have been observed in PTC, with increases in ERα, decreases in ERβ, and decreases in AR being associated with a more aggressive phenotype (Magri et al., 2012). Rajoria et al. (2010) showed that a metastatic thyroid cancer phenotype is regulated by estrogen and functional ER which enhance mitogenic, migratory, and invasive properties of thyroid cancer cells. ER and AR expression changes may be acting as a cause or a result of the onset of PTC (Magri et al., 2012). Additionally, it was previously suggested that underlying inflammatory processes may predispose healthy females toward developing thyroid cancer (Manole et al., 2001; Tafani et al., 2014).

The gender disparity in thyroid cancer incidence is a unique phenomenon, whereas nearly all other cancers without obvious anatomical bias are neutral or have a bias toward higher incidence in men. Additionally, males and females differ in their immunological responses and show unique innate and adaptive immune responses (Klein and Flanagan, 2016). It has been shown that females in general have a more active humoral and cellular immune response than men (Ansar Ahmed et al., 1985). Women are more prone to autoimmune and inflammatory thyroid diseases such as Graves’ Disease or Hashimoto’s Thyroiditis (Ngo et al., 2014). Therefore, underlying inflammatory processes in healthy females may predispose them toward developing thyroid cancer (Manole et al., 2001; Tafani et al., 2014). Despite the well-established characterization of sex disparity in thyroid cancer incidence and that inflammation plays a key role in the gender disparity of some cancers (Naugler et al., 2007), the underlying molecular factors that mediate this difference are poorly understood (Rahbari et al., 2010).

Recently, evidence has emerged showing sex differences in the expression of immune checkpoint molecules on tumors (Özdemir and Dotto, 2019; Wilms et al., 2020) and the response to checkpoint inhibition (Nosrati et al., 2017). A comprehensive analysis of the immune landscape of cancer across The Cancer Genome Atlas (TCGA) dataset specifically found the Programmed Death Receptor Ligand 1 (PD-L1) was more highly expressed among women than in men in thyroid carcinoma and three other cancer types (head and neck squamous cell, renal cell carcinoma, and lung adenocarcinoma) (Thorsson et al., 2019). Furthermore, PD-L1 has consistently been shown to be highly expressed in the most aggressive forms of thyroid cancer (Ahn et al., 2017; Chintakuntlawar et al., 2017).

Given the role gender may play in the incidence of thyroid cancer, we hypothesized that sex hormones may modulate the expression of immune checkpoint molecules, and explaining the sex disparity in their expression. In this study, we present data that suggest that AR activation attenuates PD-L1 expression in a thyroid cancer cell line, potentially by inhibiting NF-kB signaling by increasing the IkBα inhibitory subunit. Our study associates the role of androgens in the gender-specific expression patterns of PD-L1 and suggests the possibility of targeting sex hormone pathways in tandem with immunotherapeutics.

## Materials and Methods

### Patient Specimens

This study was approved by the institutional review boards of New York Eye and Ear Infirmary and New York Medical College (NYMC). Written informed consent was obtained from each patient. All TCGA data used in this study were downloaded from publicly available sources. Specimens were obtained from 44 patients undergoing thyroidectomy and fresh frozen thyroid tissue were collected between 2009 and 2013. All tumors had corresponding matched normal adjacent tissue and diagnosis of PTC was validated by pathological examination.

### RNA Sequencing

Total RNA was extracted from frozen specimens using TRIzol Reagent (Thermo Fisher Scientific, Waltham, MA, United States) and RNA cleanup was performed using RNeasy Plus Mini Kit (Qiagen, Hilden, Germany) according to manufacturer’s instructions. RNA quality, concentration, and fragment size distribution were assessed on a 2100 Bioanalyer (Agilent Inc., Palo Alto, CA, United States). Ribosomal RNA was depleted using the Illumina Ribo-Zero rRNA removal kit. Samples were aligned to the human genome using STAR software version 2.4 (Dobin et al., 2013) and quantified using RSEM version 1.2.14 (Li and Dewey, 2011). KEGG Pathway and gene ontology (GO) enrichment analyses were performed on Advaita’s iPathwayGuide^1^ Platform using DE genes [abs(log2FC) > 1.5 and *q*-value < 0.05]. Statistical tests of pathway and GO term enrichment were adjusted using FDR correction.

### TCGA Data

Level three RNA-Sequencing data from TCGA project were downloaded from the UCSC Xena Browser (Goldman et al., 2018).

### Cell Culture

The 8505C cell line was purchased from MilliporeSigma (ECACC Cat# 94090184, RRID:CVCL_1054) and the K1 cell line was graciously provided as a gift from the Schweppe lab. 84e7, K1-lentiAR, and 8505C-lentiAR cell lines were maintained in phenol-red free RPMI 1640 supplemented with 10 mM L-Glutamine, 100 U/mL penicillin/streptomycin (Corning, 30002CI, Manassas, VA, United States) and 5% United States origin, heat-inactivated fetal bovine serum (FBS) (Genesee Scientific, 25–514 H, El Cajon, CA, United States). 84e7 cells also were supplemented with 50 μg/mL G418 sulfate solution (Corning, 30–234-CR, Manassas, VA). Thyroid cancer cell line gene expression data was obtained from the Cancer Cell Line Encyclopedia (CCLE)^2^.

### Lentiviral Transduction

HEK293T cells were purchased from ATCC (ATCC Cat# CRL-3216, RRID:CVCL_0063) and cultured in DMEM supplemented with 10 mM L-Glutamine, 100 U/mL penicillin/streptomycin and 10% heat inactivated FBS. The pLENTI6.3/AR-GC-E2325 plasmid (kindly shared by the Kalland lab, Addgene plasmid # 85128^3^; RRID:Addgene_85128) along with 0.4 ug of psPAX2 packaging and 0.15 ug of VSV-G envelope plasmids were transfected into HEK293T cells using the TransIT-LT1 reagent (Mirus, MIR 2304, Madison, WI, United States). Virus-containing supernatants were collected at 48 h post-transfection and filtered using a 0.45 um PES filter. 8505C and K1 cells were treated with 40 ng/mL polybrene (MilliporeSigma #TR-1003-G, St. Louis, MO, United States) and filtered virus for 24 h followed by replacement with 10 ug/mL Blasticidin containing media. After 72 h of Blasticidin selection, cells were then sub-cultured and frozen for future experiments.

### Western Blotting

Cultured cells were lysed in RIPA Buffer (50 mM *Tris–Hcl* pH 7.4, 150 mM NaCl, 0.25% sodium deoxcholate, and 1.0% NP-40) supplemented with HALT Protease Inhibitor Cocktail (Thermo Fisher Scientific/Pierce, P/N 78430). 15 ug of total protein from each cell extract were added to 5 uL of sample buffer (10% β-mercaptoethanol in 4× Laemmli buffer) followed by SDS-PAGE electrophoresis performed under reducing conditions with a 4% stacking gel and 10% separating gel. Proteins were transferred from the gel to an Immobilon-P PVDF Membrane (MilliporeSigma, P/N IPVH00010). Membranes were incubated with primary antibodies diluted in 5% milk (total protein) or 1% milk (phosphorylated protein) in 1× TBST overnight at 4°C. Secondary goat anti-rabbit Horseradish Peroxidase (HRP) conjugated secondary antibody (Abcam Cat# ab6721, RRID:AB_955447) was diluted 1:5,000 in 5% milk with 1× TBST and incubated with membrane for 2 h at room temperature. Quantitative analysis of optical density was performed with ImageJ. Antibodies used for western blotting can be found in Supplementary Table 1.

### Flow Cytometry

A 0.5–1 × 10^6^ cultured cells were collected using cell scrapers and resuspended in 100 μL incubation buffer (0.25 g bovine serum albumin dissolved in 50 mL 1 × PBS) with 20 μL of FITC-conjugated mouse anti-human primary PD-L1 monoclonal antibody (BD Pharmingen, P/N 558065) for 1 h at room temperature. Cells were rinsed 2× in incubation buffer and finally resuspended in 0.45 mL ice cold 1× PBS and strained into polystyrene flow cytometry tubes. Flow Cytometry was conducted using the BD FACScan Flow Cytometer and each experiment was conducted in triplicate. Examples for gating parameters can be found in Supplementary Figure 1. Relative fluorescence intensity (RFI) was calculated by normalizing mean fluorescence intensity (MFI) measurements of treated samples to vehicle control:

(1) $$RFI=\frac{MFI_{treatment}}{MFI_{control}}$$

### Cleavage Under Targets and Tagmentation (CUT&Tag) and Sequencing

For CUT&Tag, 10^6^ 8505C-AR cells treated with either DHT or vehicle control for 48 h were used. CUT&Tag DNA were prepared according to Kaya-Okur et al. (2019). The AR primary Rabbit mAb (Cell Signaling #5153) and Guinea Pig anti-Rabbit secondary antibody (Novus Biologicals #NBP1-72763, Centennial, CO, United States) were used as well as the CUTANA^TM^ pAG-Tn5 (Epicypher #15-1017, Durham, NC, United States).

Samples were multiplexed and sequenced on a Nextseq 500 in paired-end mode (38/37) with a high-output, 75-cycle reagent kit. 3-prime read ends containing Nextera Mate Pair adapters were trimmed using cutadapt (Martin, 2011), filtering reads < 20 bp (-m 20) or with an average phred quality score < 20 (-q 20). Trimmed FASTQ files were aligned to the hg38 genome assembly using BWA mem (Li, 2013), marking duplicate reads (-M). Aligned SAM files were compressed to BAM format, sorted, merged, and indexed using samtools (Li et al., 2009). Duplicate reads were removed from BAM files using Picard Toolkit MarkDuplicates (Broad Institute, 2019).

To compare AR binding of genomic loci across biological conditions, we first used deeptools bamCoverage (Ramírez et al., 2014) to generate normalized BigWig files from de-duplicated BAMs. Parameters selected include counts-per-million normalization (–normalizeUsing CPM) with a bin size of 10 bp (-*bs* = 10). Next, we calculated the aggregate normalized accessibility signal at known murine adult *cis*-regulatory elements (CREs) using UCSC bigWigAverageOverBed; these elements are subclassified as promoters, proximal enhancers and distal enhancers, and assigned a unique accession by ENCODE (ENCODE Project Consortium Moore et al., 2020). We used a Z-scoring method to identify high-confidence AR peaks in promoters. Total signal across *cis*-regulatory elements was recorded for each replicate using bigWigAverageOverBed. Only elements ranked in the top 10 percent (*Z* > 1.64) by signal were retained. The intersect of each replicate’s top elements constitutes our high-confidence element list.

### Statistical Analysis

Data analysis was performed on Prism version 8.1.2 (GraphPad Software, San Diego, CA, United States) and R version 3.6.2. Unless otherwise indicated, all experimental data were expressed as the mean ± SD of experiments performed in triplicate. For analysis of paired NYMC tumor and normal tissue, a two-tailed, paired Student’s *t*-test was performed. For comparison of tumor and normal TCGA data, a two-tailed Welch’s *t*-test was used for statistical analysis. For all culture experiments, statistical significance of results was analyzed by two-tailed, and paired Student *t*-tests. For immunohistochemistry (IHC), Fisher’s exact test was performed.

## Results

### Inflammation and AR Expression in Male and Female PTC

To assess the difference in inflammatory and immune responses between male and female thyroid cancer, we performed RNA-sequencing on 44 PTC with matched-paired normal thyroid tissue in the NYMC biobank (33 female and 11 male). A total of 1532 protein-coding genes and 756 non-coding RNAs underwent ≥ 1.5-fold change between tumor and matched normal tissue (*q*-value ≤ 0.05). Principal component analysis (PCA) on all specimens demonstrated distinct separation on the PC2 axis between PTC and normal adjacent tissue, indicating that 10% of transcriptomic variance was sufficient to distinguish tumor samples from normal tissue (Supplementary Figure 2A). KEGG pathway enrichment analysis of differentially expressed genes (DEGs) identified cell adhesion molecules and ECM-receptor interaction as well as pathways related to host immune function (Cytokine-cytokine receptor interactions and allograft rejection) (Supplementary Figure 2B). These findings are consistent with previous transcriptomic studies of PTC (Song et al., 2018).

**FIGURE 1:**
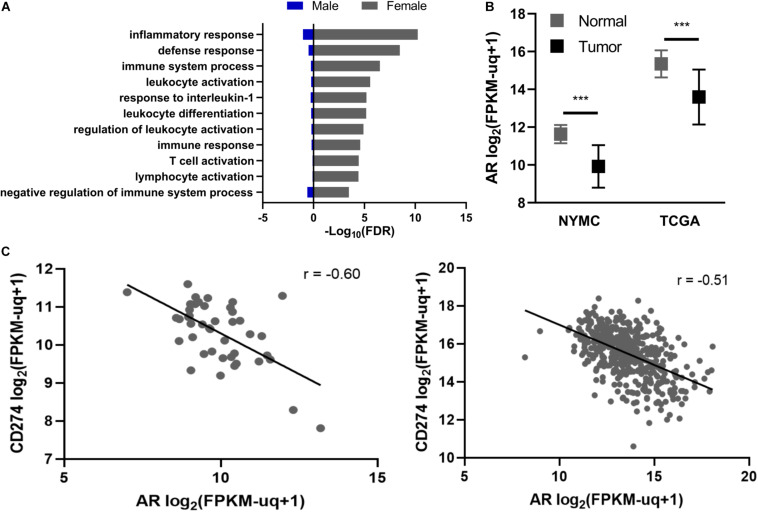
Relationship between androgen receptor (AR), gender, and *CD274* expression. **(A)** Enrichment of GO biological processes incorporating inflammation and immune responses. **(B)** AR expression in primary PTC tissue (Tumor) vs. normal adjacent thyroid tissue (Normal) in the New York Medical College (NYMC) and The Cancer Genome Atlas (TCGA) datasets. AR expression was compared using Student’s and Welch’s paired *t*-tests for the NYMC and TCGA datasets, respectively. **(C)** Regression analysis of *CD274* and AR mRNA levels in NYMC (left) and TCGA (right) tumors. r represents Pearson’s correlation coefficient. FPKM-UQ, Fragments per kilobase million-upper quartile normalized. ***p < 0.0005.

Next, we examined GO terms enriched in DEGs from male and female PTC. A subset of GO biological processes related to adaptive immunity and inflammation were significantly enriched among female PTC but not male PTC (Figure 1A). Furthermore, genes that mediate T-cell activation such as CD40L, ICOS, and IL-2 were downregulated in female PTC (Supplementary Figure 3A).

Given the distinct gene expression patterns among immune-related genes between female and male thyroid tissue, we next examined the expression of AR and its relationship to these gene expression patterns. AR expression in both the NYMC RNA-seq dataset and TCGA Thyroid Carcinoma project’s RNA-sequencing dataset showed significant (*p* < 0.0001) reduction in AR expression in tumors compared to normal tissue (Figure 1B). We performed a co-expression analysis between AR and a set of immune regulatory molecules, including CD28, CD80, CD86, CTLA-4, PD-1, PD-L2, TIM-3, TIM-3L, 4-1BB, 4-1BBL, OX40, and OX40L, in the NYMC dataset and found the expression of the immune checkpoint molecule *CD274* had a significant inverse correlation (Pearson rho = -0.60) with AR and this finding was replicated with TCGA data (Figure 1C). Furthermore, males have a higher rate (70%) of exhibiting an inverse relationship between AR and PD-L1 than females (35%) (*p* < 0.05, Supplementary Figure 3B).

### Androgen Downregulates PD-L1 in an AR-Dependent Mechanism

**FIGURE 2:**
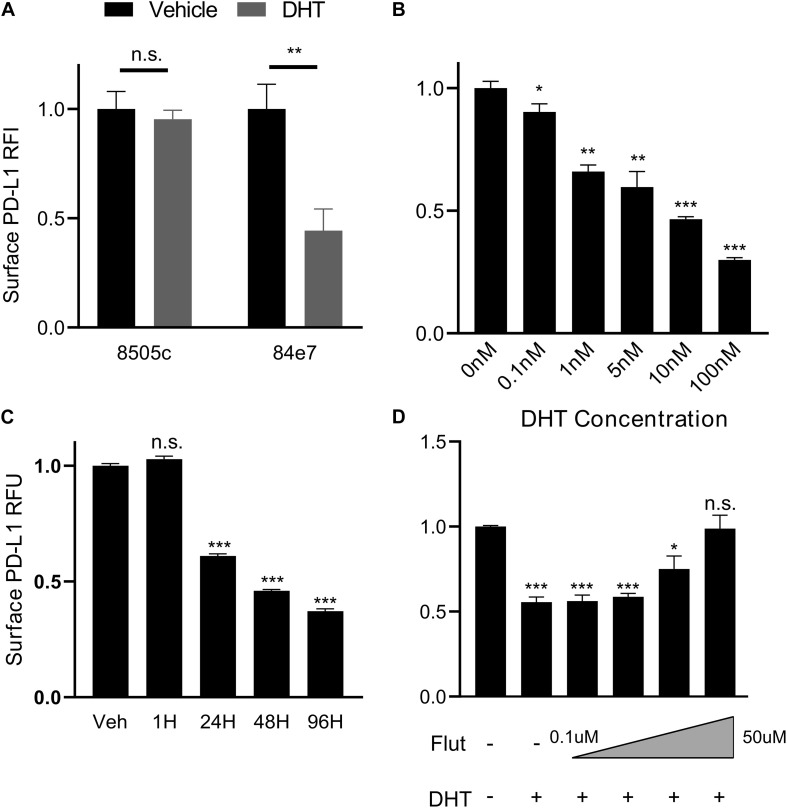
PD-L1 is downregulated by androgen in an AR-dependent manner. **(A)** Surface expression of PD-L1 determined by flow cytometry in AR-lacking (8505C) or AR-expressing (84E7) thyroid cancer cell line after treatment with Vehicle (ethanol) or Dihydrotestosterone (DHT) (10 nM) for 72 h. **(B)** Dose-response to variable DHT concentrations in 84E7 cells at 72 h. **(C)** Time course of variable treatment lengths with 10 nM DHT. **(D)** Surface PD-L1 expression in 84E7 cell lines treated with 10 uM DHT and increasing concentrations of flutamide (Flut) at concentrations of 0.1, 1, 10, and 50 uM. RFI, relative fluorescence intensity. Student *t*-tests paired to control conditions *p < 0.05; **p < 0.005; and ***p < 0.0005. Barplots represents mean ± SEM of experiments performed in triplicate. *ns*, not significant.

Given the correlation between PD-L1 and AR expression in primary PTC tissue, we designed a series of cell culture experiments to test whether androgens regulate the expression of PD-L1 in thyroid cancer. The undifferentiated thyroid cancer cell line, 8505C, was used due to the high surface level expression of PD-L1 (Cantara et al., 2019) and higher AR expression relative to other thyroid cancer cell lines based on mRNA-sequencing and protein array data in the CCLE (Supplementary Figure 4). However, androgen treatment of the 8505C cell line failed to increase expression of the AR-responsive gene FKBP5 (Supplementary Figure 6A). We therefore used a transfected clone, termed 84e7 (Jones et al., 2021), that constitutively expresses human AR in a pcDNA3.1 vector under a CMV promoter. We initially measured PD-L1 levels by Western blot and detected an approximately 30% reduction (*p* < 0.01) in PD-L1 expression after 72 h of treatment with the potent androgen DHT but no change at 24 h (Supplementary Figure 5). Next, we quantified surface expression of PD-L1 using flow cytometry in 8505C and 84e7 cells treated with DHT or vehicle control at the 72 h timepoint. Cell surface PD-L1 levels decreased in 10 nM DHT-treated 84e7 by approximately 60% (*p* < 0.005) as compared to vehicle-treated 84e7 while no change was observed between vehicle- and DHT-treated 8505C (Figure 2A). Notably, the DHT-mediated downregulation of PD-L1 was replicated in another thyroid cancer cell line, K1, and stably expressing the pLENTI6.3/AR-GC-E2325 construct (Supplementary Figure 6B). A dose response curve that encompassed all human physiological ranges of total androgens was used, from 0.1 nM which is five times below the low end of the female physiological range, to 100 nM which is three times above the high end of the male physiological range (Salameh et al., 2010; Figure 2B). Interestingly, the greatest impact of DHT occurred in the male physiological range, with a 50% reduction in PD-L1 expression as compared to the female dose range. Next, we performed a time course of 10 nM DHT treatment in 84e7 cells and found that the 60% reduction in PD-L1 surface expression was achieved by 48 h of treatment (Figure 2C).

To confirm that the cell surface decrease in PD-L1 observed in DHT-treated 84e7 was specific to AR signaling, 8505C, and 84e7 were treated with varying concentrations of flutamide, a selective AR antagonist (Goldspiel and Kohler, 1990), prior to DHT treatment to pharmacologically inhibit AR-induced signaling. Flutamide was shown to inhibit the ability of DHT to decrease PD-L1 in 84e7, resulting in a dose-dependent and > 90% restoration of cell surface PD-L1 expression at 50 uM concentration (Figure 2D), but not in 8505C cells (Supplementary Figure 6C) indicating that AR activity is necessary for DHT-mediated PD-L1 downregulation.

### CUT&Tag Identifies AR Binding to Regulators of PD-L1

**FIGURE 3:**
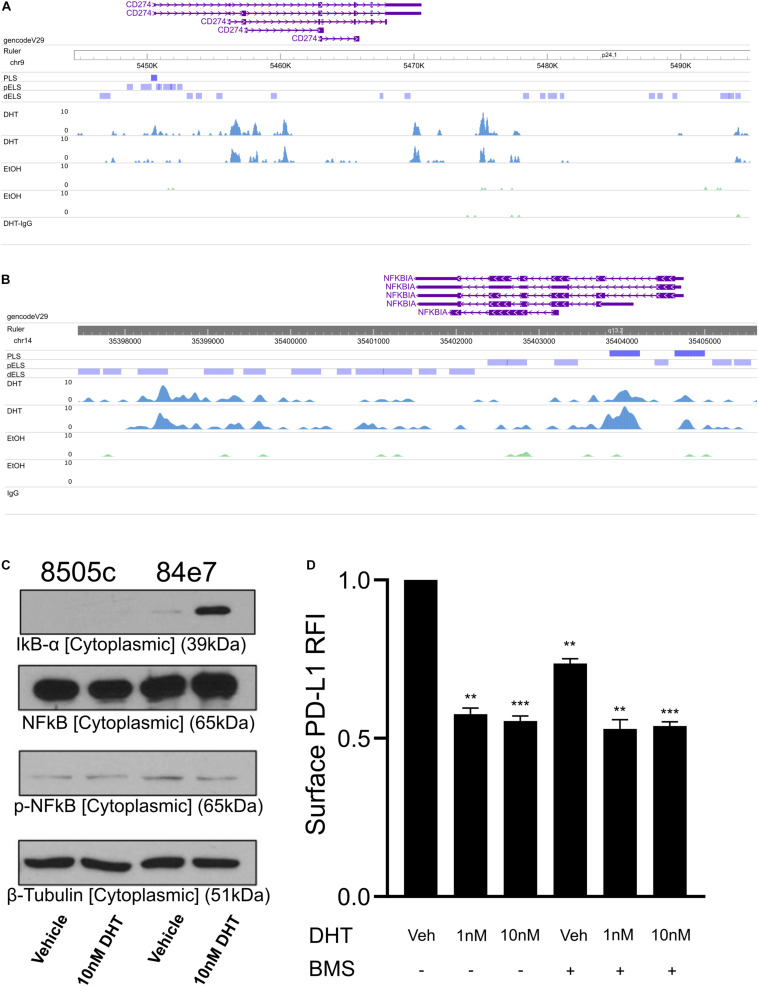
Profiling AR binding genome-wide in 8505C-AR cells. CUT&Tag AR signal at the *CD274* gene body **(A)** and the NFKBIA gene promoter **(B)**. **(C)** Western blot analysis of cell cytoplasmic fraction from 72 h vehicle and 10 nM DHT-treated 8505C and 84E7. **(D)** Surface PD-L1 expression in 84E7 cells co-treated with 10 nM DHT for 48 h and the IKK inhibitor BMS-345541 (BMS) at 10 μM for 24 h. PLS, promoter-like signal; pELS, predicted enhancer-like signal; IgG, isotype control IgG CUT&Tag. Student *t*-tests paired to control conditions **p < 0.005 and ***p < 0.0005.

To begin identifying potential mechanisms by which androgen signaling downregulates PD-L1 expression, we profiled AR binding genome wide using cleavage under targeted nuclease and tagmentation (CUT&Tag) (Kaya-Okur et al., 2019). Since the 84e7 cell line required growth media supplemented with the G418 antibiotic that could confound results of genome-wide sequencing experiments, we generated an 8505C cell line stably expressing AR without requiring constant antibiotic selection via lentiviral transduction (Ryu et al., 2017). CUT&Tag sequencing of this cell line (named 8505C-lentiAR) after treatment with 10 nM DHT for 48 h revealed direct AR binding to the PD-L1 gene body (Figure 3A), as well as potential regulatory elements downstream of the PD-L1 open reading frame.

PD-L1 expression is regulated by various signaling pathways including NFκB, MAPK, PI3K, mTOR, and JAK/STAT (Ritprajak and Azuma, 2015). We therefore surveyed our AR binding sites for promoter binding to molecules involved in these pathways and identified an AR binding signal at the NFKBIA promoter (Figure 3B). The NFKBIA gene encodes the IkBα protein that sequesters NFkB in the cytoplasm, reducing NF-kB translocation to the nucleus. We confirmed that DHT increased both total and cytoplasmic IkBα proteins levels via western blot (Figure 3C and Supplementary Figure 7).

We next aimed to determine if androgen-mediated IkBα upregulation contributes to the downregulation of PD-L1. 84e7 cells were treated with a selective and potent inhibitor of IKK, BMS-345541. Inhibition of IKK maintains IkBα in the unphosphorylated state, resulting in sequestration of NF-κB in the cytoplasm (Burke et al., 2003). PD-L1 levels in 84e7 treated with BMS-345541 decreased significantly, but did not decrease PD-L1 surface expression to the same extent as 1 nM or 10 nM DHT (Figure 3D). When combined with DHT, there was no additive or synergistic effect on PD-L1 levels (Figure 3D). Thus, inhibition of IKK does indeed impact PD-L1 expression but does not account entirely for the decrease in PD-L1 observed with DHT, supporting a model where PD-L1 expression is modulated via AR activity at multiple regulatory elements.

## Discussion

Despite the fact that thyroid cancer has a relatively quiet mutagenic landscape (Lawrence et al., 2013), there is increasing evidence that immune checkpoint molecules, specifically PD-L1, and play a role in thyroid cancer pathogenesis (Chowdhury et al., 2016; Chintakuntlawar et al., 2017). Inflamed thyroid follicular cells express PD-L1 (Álvarez-Sierra et al., 2019) and immune checkpoint induced thyrotoxicity is a common adverse effect of PD1/PD-L1 blockade (D’Andréa et al., 2021). Furthermore, PD-L1 inhibitor-induced thyroiditis has been shown to be an indicator of the drug’s efficacy (Kotwal et al., 2020), suggesting functional PD-L1 expression in native thyroid glands. The expression of PD-L1 in thyroid cancers has been extensively studied using IHC (Aghajani et al., 2018). PD-L1 positivity is correlated with lower overall survival in anaplastic thyroid cancer (Chintakuntlawar et al., 2017) and worse clinicopathologic characteristics in PTC including reduced progression-free survival, increased lymphovascular invasion, tumor size, and TNM stage (Chowdhury et al., 2016; Shi et al., 2017; Gillanders and O’Neill, 2018). While the prevalence and severity of thyroid cancer differs between males and females, PD-L1 IHC in PTC exhibits no sex-based changes in the percentage of positively staining cells (Aghajani et al., 2018). The bulk RNA-sequencing performed in this study aggregates PD-L1 expression in the entire tissue while IHC indicates a proportion of cells that express PD-L1 without providing the degree of expression for the individual cell. Furthermore, our data indicates that AR is significantly downregulated in both male and female thyroid tumors compared to normal tissues. This may partially explain why PD-L1 IHC shows no sex-related differences as androgen-mediated PD-L1 attenuation is blunted by the downregulation of AR in male tumors.

To our knowledge, this is the first study identifying a relationship between AR activity and PD-L1 expression in thyroid cancer. Recently, Jiang et al. (2020) characterized AR suppression of PD-L1 in hepatocellular carcinoma. In combination, these findings point to a previously unappreciated role AR has on PD-L1 expression in multiple tissue types and, given that the PD-1/PD-L1 axis is an actionable immunotherapeutic target for various cancers, indicate that AR activity can impact the response to immunotherapy. An ongoing phase II clinical trial is investigating the efficacy of atezolizumab, a humanized PD-L1 monoclonal antibody, in combination with small molecule inhibitors or cytotoxic agents in the most aggressive forms of thyroid cancer (NCT03181100). Preliminary results for this trial were recently reported at the 2020 ASCO Annual Meeting and showed that compared to the historical ATC OS median of 5 months, patients in all three atezolizumab cohorts had a median OS of over 18 months. Overall, 10 of 38 patients had complete tumor resection after therapy and seven of those patients were alive at the time of the report (Cabanillas et al., 2020).

Similar to Jiang et al. (2020), we identified a direct interaction between AR and the *CD274* gene body using CUT&Tag. However, since we were able to profile AR binding sites genome-wide, we also identified an AR binding signal at the NFKBIA promoter. AR activation may impact NF-kB signaling by increasing IkBα which thereby prevents NF-kB translocation into the nucleus. A decrease in nuclear NF-kB results in a direct reduction in PD-L1 expression due to the complex’s involvement in key enhancer-promoter interactions (Chen et al., 2018). However, inhibition of NF-kB signaling using BMS-345541 did not reduce PD-L1 expression to the same level as DHT, suggesting AR likely represses PD-L1 via multiple mechanisms. This may include direct inhibition of PD-L1 promoter and enhancer elements as well as modulation of signaling pathways responsible for regulating PD-L1 expression, such as the IFNγ pathway.

There is a well-established gender disparity in thyroid cancer incidence. Underlying inflammatory processes in healthy females may predispose them toward developing thyroid cancer (Manole et al., 2001; Tafani et al., 2014). Klein and Flanagan (2016) have stated that sex is a biological variable that should be considered in immunological studies. We postulate that reduced levels of PD-L1 in males allows for continuous immunosurveillance and elimination of nascent tumors, whereas higher levels of PD-L1 in females results in immuno-evasive phenotypes (Mould et al., 2017). In addition to the role of AR in modulating the PD-L1 expression, ERα activation by 17β-Estradiol upregulates PD-L1 protein expression, and perhaps contributing to the immune inhibitory environment in women (Yang et al., 2017). Given the gender difference in inflammatory thyroiditis, which can contribute to elevated PD-L1 levels due to expression of cytokines such as IFNγ, constitutively active immunological processes, lower AR levels and/or estrogen receptor activation in females may lead to elevated PD-L1 levels, thereby contributing to the thyroid cancer gender disparity. However, the present study is limited to only providing correlative data in primary human thyroid carcinomas to suggest the relationship between AR activity and PD-L1. Furthermore, our cell culture model is limited by the use of exogenous expression of AR in an immortalized thyroid cancer cell line which may not represent the physiologic role of AR signaling *in vivo*. While the 8505C cell line expresses relatively high levels of AR compared to other thyroid cancer cell lines, it is evident from our results that these cells do not respond to DHT stimulation. Further investigation into the role of AR in primary thyroid cancer cells, particularly PTC cells, is warranted to establish a potential underlying mechanism and its role in the disparity in incidence.

In summary, this study suggests that PD-L1 is downregulated by androgens in thyroid cancer cells in an AR-dependent mechanism. This putative mechanism is corroborated by the inverse correlation of AR and PD-L1 expression in primary human tissues and the higher frequency of PD-L1 expression in female PTC tissues. Genome-wide profiling of AR binding sites reveals both direct activity at the PD-L1 locus as well as indirect regulatory mechanisms via inhibition of NF-kB through upregulation of IkBα. These findings provide a foundation for investigating the role PD-L1 expression has in the pathogenesis of thyroid cancer and the disparity in incidence between the genders.

## Data Availability Statement

The datasets presented in this study can be found in online repositories. The names of the repository/repositories and accession number(s) can be found below: https://www.ncbi.nlm.nih.gov/bioproject/?term=PRJNA698545, PRJNA698545.

## Ethics Statement

The studies involving human participants were reviewed and approved by the Institutional Review Board at New York Medical College, New York Eye, and Ear Infirmary. The patients/participants provided their written informed consent to participate in this study.

## Author Contributions

TO’C, RKT, and JG conceived of the study and designed the experiments. TO’C, SD, AG, and MJ performed the experiments. TO’C, SD, and TR analyzed and interpreted the data. ES, AM, CI, and HI provided clinical expertise and primary tissue. TO’C and SD wrote the manuscript. All authors contributed to the article and approved the submitted version.

## Conflict of Interest

The authors declare that the research was conducted in the absence of any commercial or financial relationships that could be construed as a potential conflict of interest.

## Publisher’s Note

All claims expressed in this article are solely those of the authors and do not necessarily represent those of their affiliated organizations, or those of the publisher, the editors and the reviewers. Any product that may be evaluated in this article, or claim that may be made by its manufacturer, is not guaranteed or endorsed by the publisher.

## Footnotes

1
http://www.advaitabio.com/ipathwayguide

2
http://www.broadinstitute.org/ccle/home

3
http://n2t.net/addgene:85128

## Supplementary Material

Supplementary Table 1 Primary antibodies used for western blotting.
