# Supplementary material for: Androgen Activity Is Associated With PD-L1 Downregulation in Thyroid Cancer
Source: Front Cell Dev Biol. 2021 Aug 6;9:663130. doi: 10.3389/fcell.2021.663130 (PMC8377372; doi:10.3389/fcell.2021.663130)
Supplement: Supplementary Table 1 — Primary antibodies used for western blotting. [file Table_1.pdf]

**Antibody**	**Isotype**	**Total / Phospho**	**Poly/mAb**	**Size (kDa)**	**Manufacturer**	**P/N**	**Dilution**	**RRID**
PD-L1	Rabbit IgG	Total	Poly	33	ThermoFisher	PA5-20343	1:500	AB_11153819
NFkB (p65)	Rabbit IgG	Total	mAb	65	CST	8242	1:1000	AB_10859369
p-NFkB (p65/Ser536)	Rabbit IgG	Phospho	mAb	65	CST	3033	1:500	AB_331284
IkBα	Rabbit IgG	Total	mAb	39	CST	4812	1:500	AB_10694416
α-Tubulin	Rabbit IgG	Total	mAb	52	CST	2125	1:1000	AB_2619646
β-Tubulin	Rabbit IgG	Total	mAb	55	CST	2128	1:1000	AB_823664
