# Supplementary material for: Androgen Activity Is Associated With PD-L1 Downregulation in Thyroid Cancer
Source: Front Cell Dev Biol. 2021 Aug 6;9:663130. doi: 10.3389/fcell.2021.663130 (PMC8377372; doi:10.3389/fcell.2021.663130)
Supplement: Supplementary file 2 [file Data_Sheet_1.PDF]

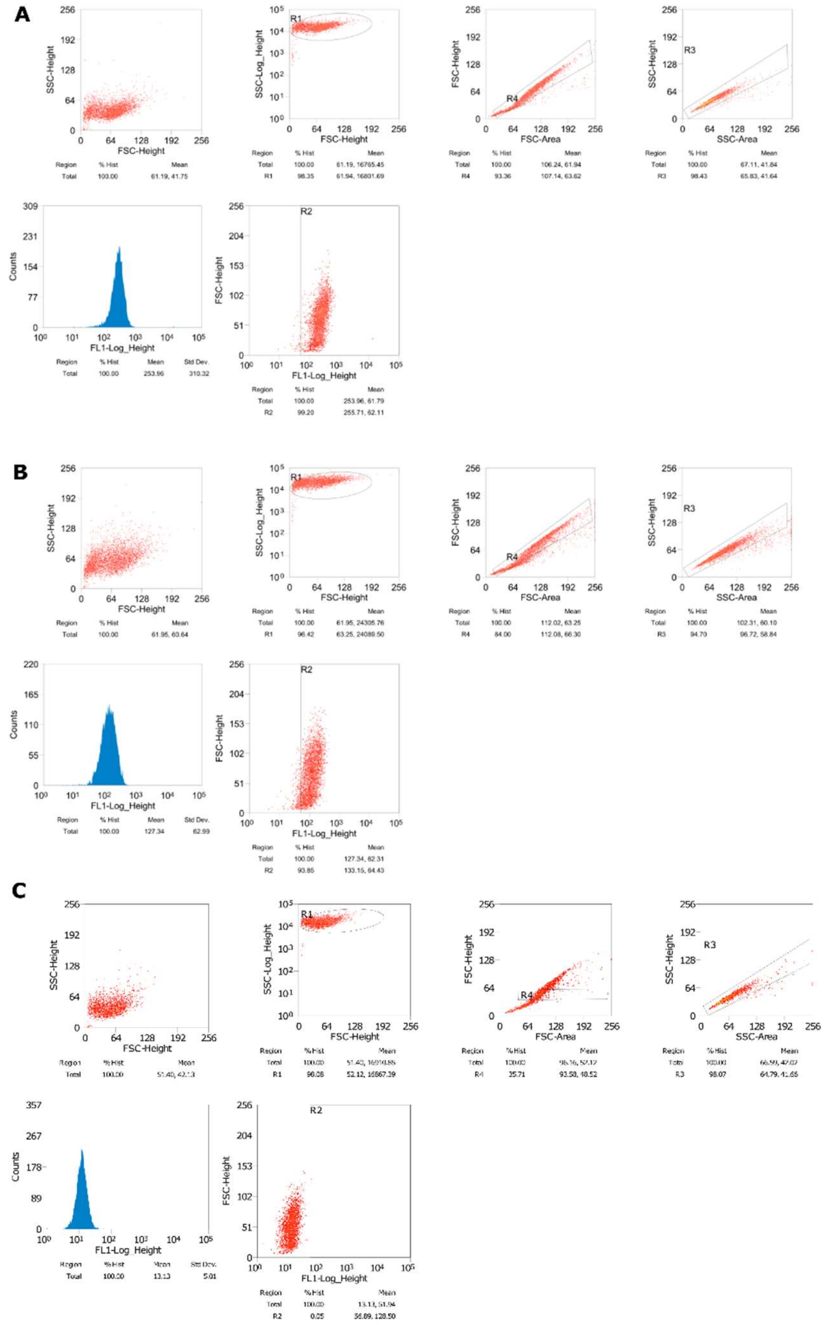

Supplemental Figure 1: Representative gating parameters for flow cytometry experiments in 84e7 cells treated with vehicle (A), DHT (B) or unstained (C). SSC, side scatter; FSC, forward scatter; FL1, FITC signal.

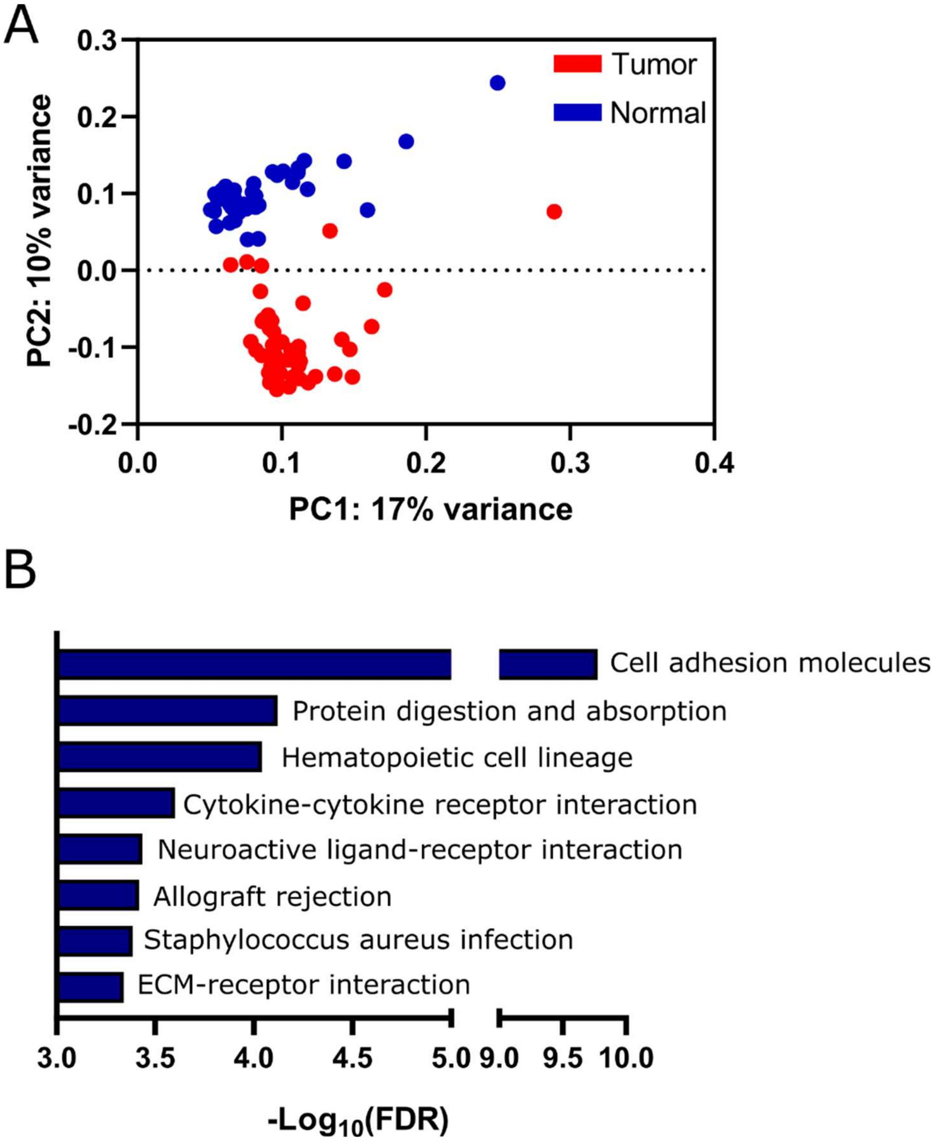

Supplemental Figure 2: Differential expression analysis between tumor and normal thyroid tissue. (**A**) Principal component analysis of 44 PTC and matched normal tissue. Each point represents an individual tumor (red) or normal (blue) sample. (**B**) Top 8 KEGG Pathway analysis of differentially expressed genes. PC, principal component; FDR, false discovery rate.

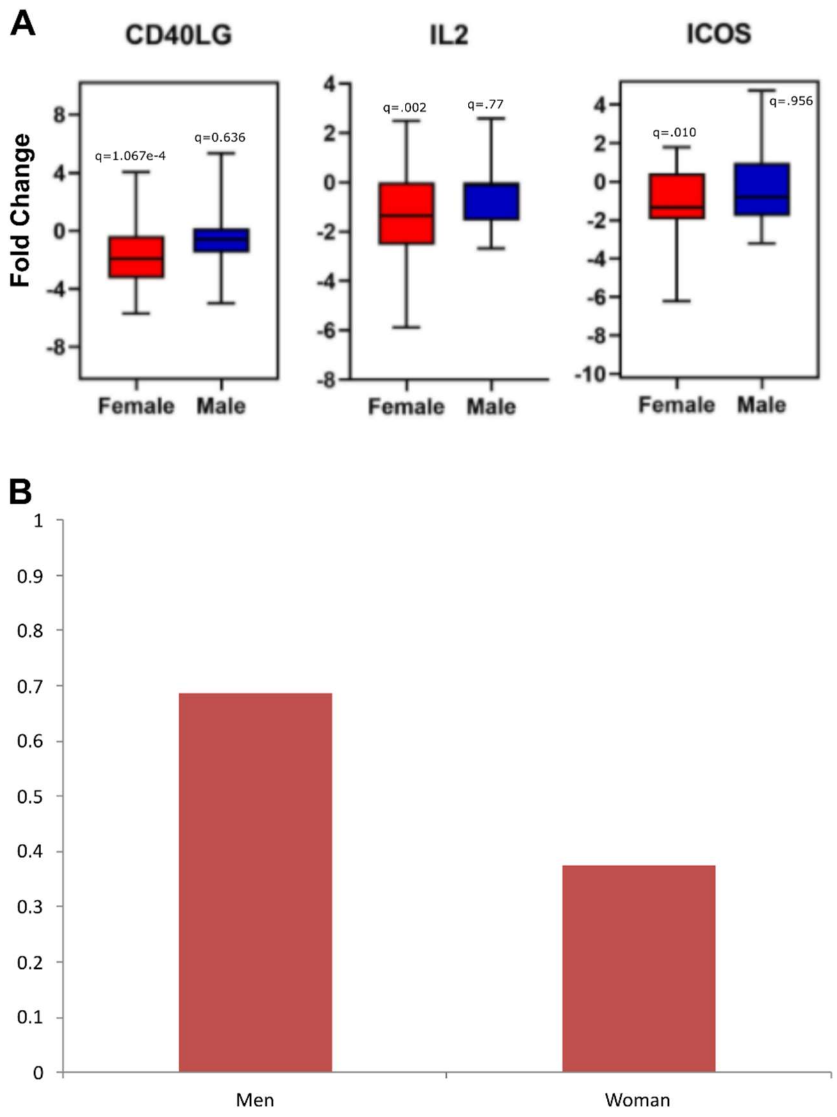

Supplemental Figure 3: (**A**) log 2 (Fold Change) between tumor and normal tissue of 3 key T-cell activating molecules (CD40LG, IL2, ICOS) shows downregulation in females but not males. Q-values represent two-sided paired t-test p-values adjusted for multiple comparisons. (**B**) Percentage of patients in the TCGA data with inverse relationship between AR and PD-L1 fold change between tumor and normal tissue (n = 55).

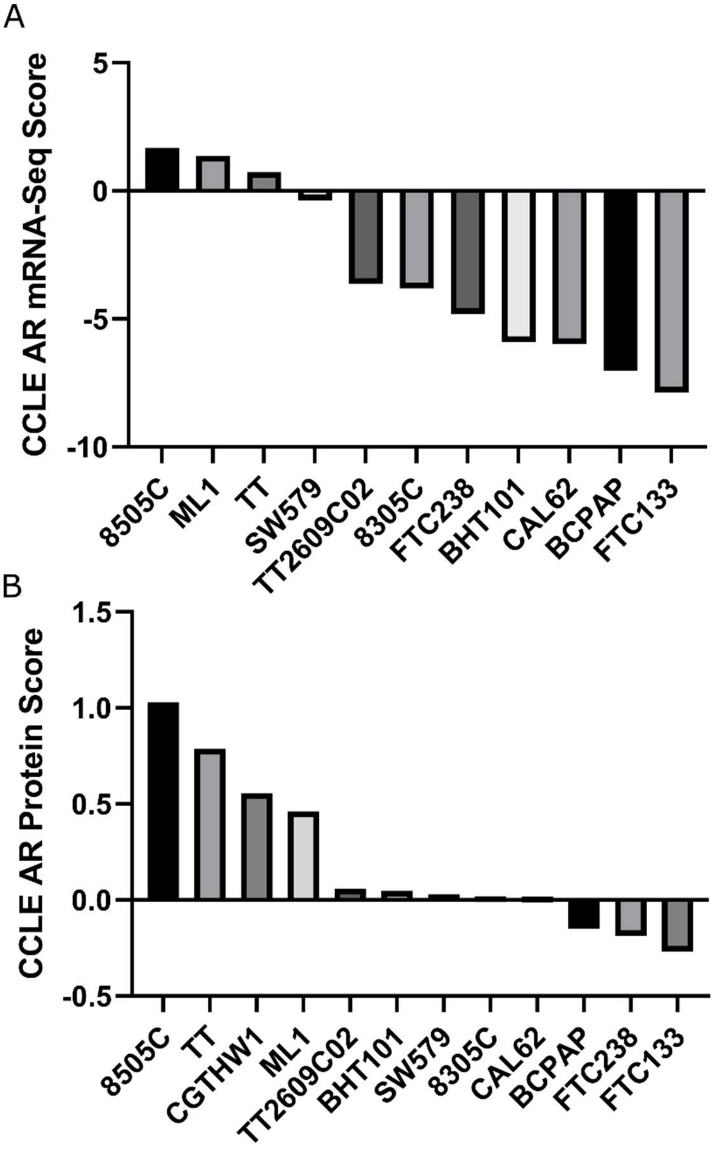

Supplemental Figure 4: mRNA (**A**) and protein (**B**) expression of AR across thyroid cancer cell lines in the Cancer Cell Line Encyclopedia (CCLE). Data was obtained from the CCLE website (https://portals.broadinstitute.org/ccle) and represent single experiments for each cell line.

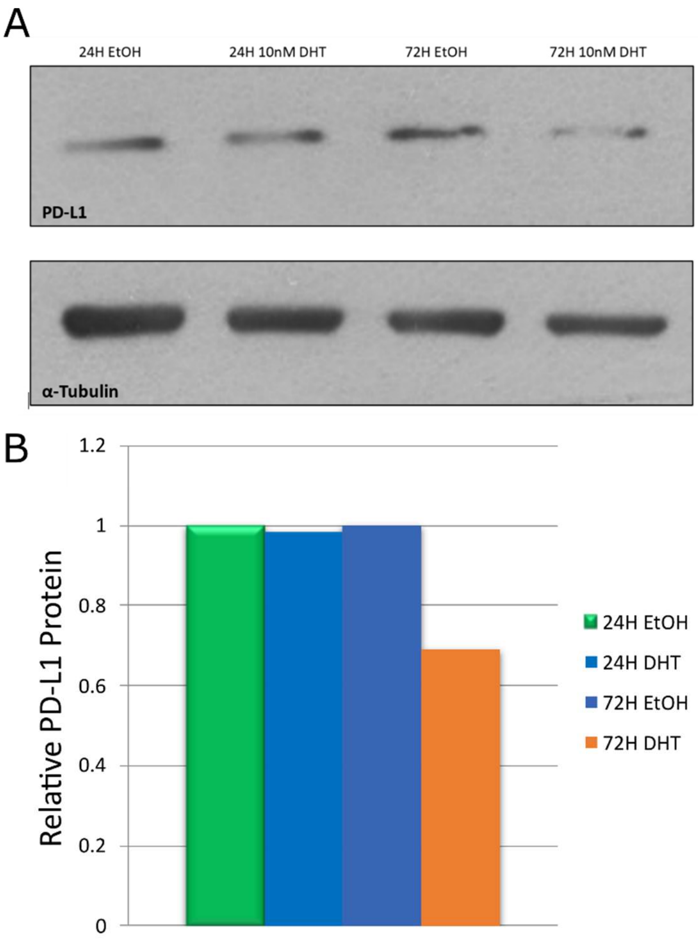

Supplemental Figure 5: Western blot analysis of PD-L1 expression using total protein lysates from 84e7 cells. (**A**) Representative western blot and quantification (**B**) using intensity (gray value) of PD-L1 band normalized to α-Tubulin (n = 3 per treatment).

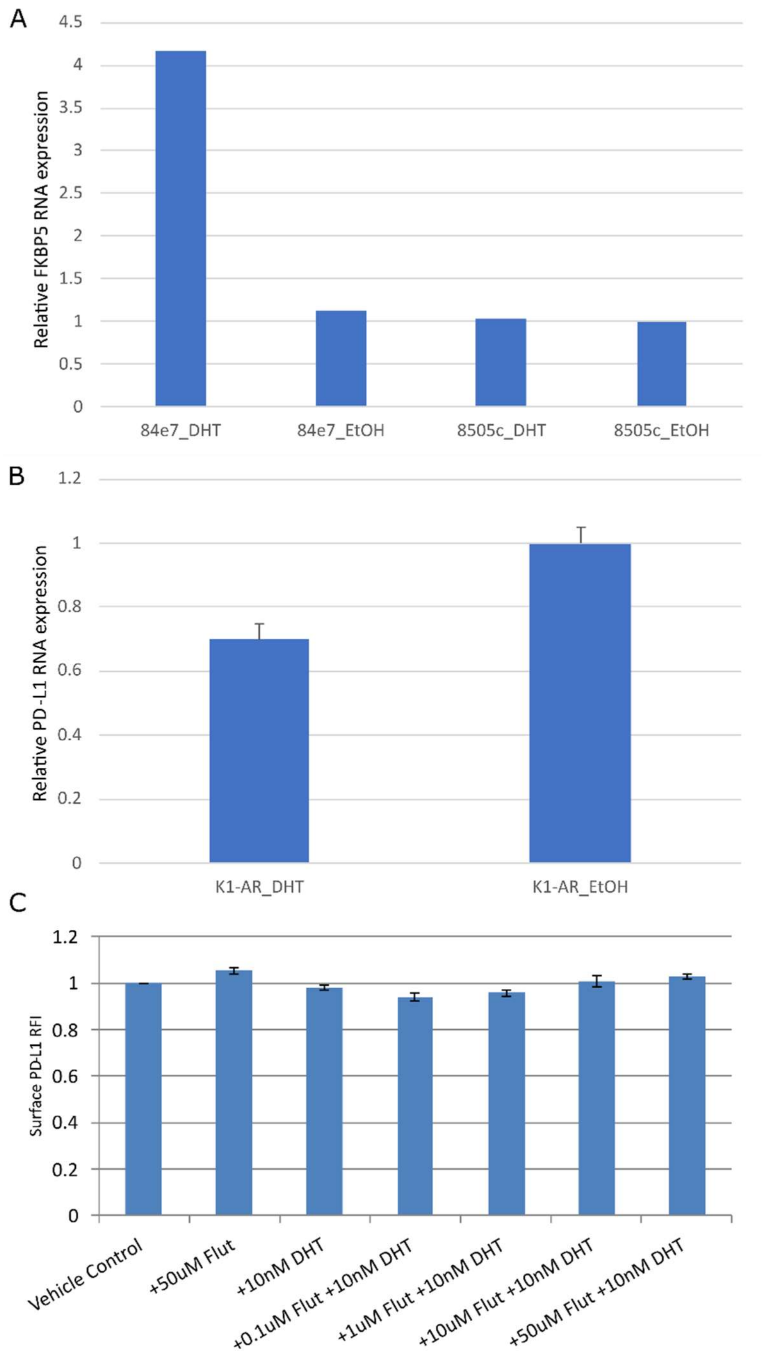

Supplemental Figure 6: (**A**) Expression of the AR-responsive gene FKBP5 in 8505C and 84e7 cells treated with 10nM DHT or vehicle control (EtOH). (**B**) PD-L1 expression in K1 cells transduced with AR lentivector (K1-AR) treated with 10nM DHT or vehicle control. Cell were treated for 48hrs and mRNA quantified using RT-qPCR in triplicate. (**C**) Surface PD-L1 expression in 8505C cell lines treated with DHT and increasing concentrations of flutamide (Flut). RFI, relative fluorescence intensity.

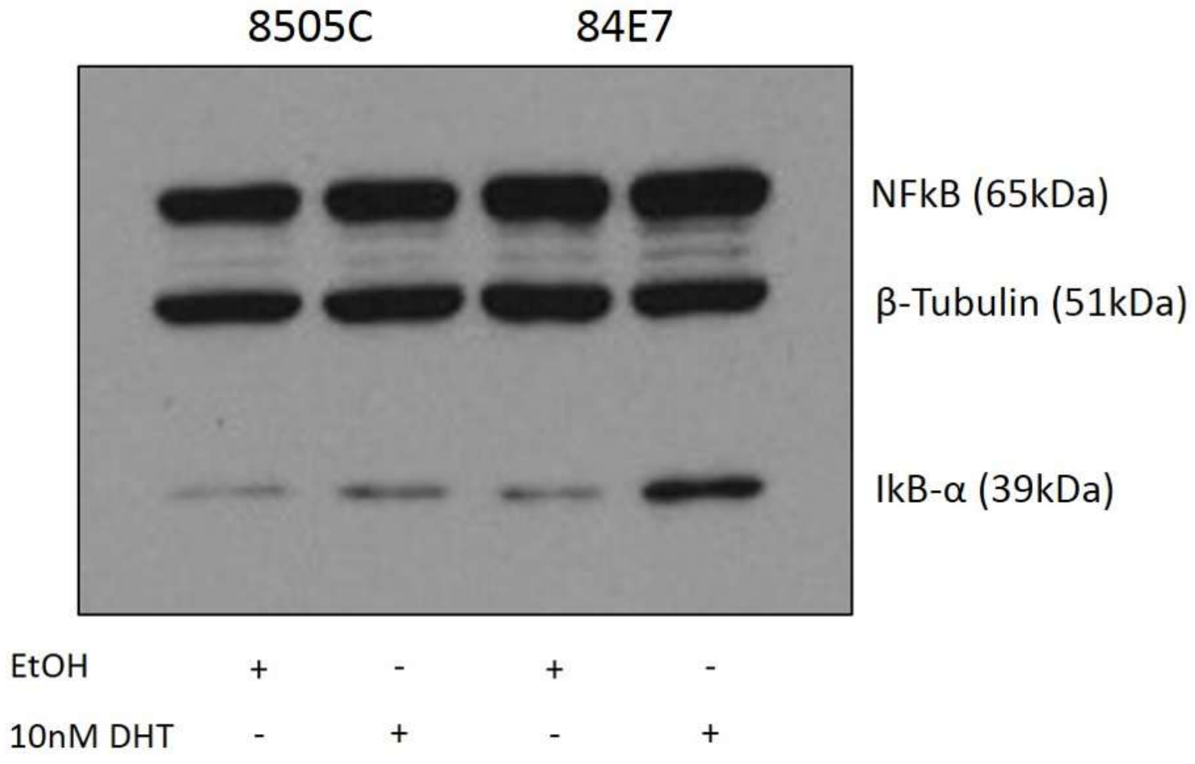

Supplemental Figure 7: Total protein western blot of 8505C and 84e7 cell lines treated with 10nM DHT or vehicle control (EtOH) for 72hrs measuring NFkB and IkB-α.
